# Human papillomavirus (HPV) screening and cervical cancer burden. A Brazilian perspective

**DOI:** 10.1186/s12985-015-0342-0

**Published:** 2015-07-25

**Authors:** Adriana T. Lorenzi, Kari J. Syrjänen, Adhemar Longatto-Filho

**Affiliations:** Molecular Oncology Center, Barretos Cancer Hospital, Barretos, Brazil; Biohit HealthCare Oyj, Helsinki, Finland; Cancer Prevention Department, Barretos Cancer Hospital, Barretos, Brazil; Laboratory of Medical Investigation (LIM) 14, Faculty of Medicine, São Paulo University, São Paulo, Brazil; Life and Health Sciences Research Institute, ICVS, School of Health Sciences, Minho University, Braga, Portugal; ICVS/3B’s – PT Government Associate laboratory, Braga, Portugal

**Keywords:** Cervical cancer, HPV DNA test, Molecular diagnostic techniques, Cancer screening, Pap test

## Abstract

This review tackles the issues related to disease burden caused by cervical cancer (CC) and its precursor (CIN) lesions in Brazil. A special focus is given to new technologies with potential to interfere with the development of CC by reducing the high-risk human papillomavirus (hr-HPV)-induced lesions that remain a major public health burden in all developing countries where organized screening programs do not exist. Globally, 85 % of all incident CC and 50 % of CC deaths occur in the developing countries. Unfortunately, most regions of Brazil still demonstrate high mortality rates, ranking CC as the second most common cancer among Brazilian women. Recently, CC screening programs have been tailored in the country to enable early detection of CC precursor lesions and thereby reduce cancer mortality. A combination of HPV testing with liquid-based cytology (LBC) seems to be a promising new approach in CC screening, with high expectation to offer an adequate control of CC burden in this country.

## Introduction

### Short overview on human papillomavirus (HPV)

Human papillomavirus (HPV) is a non-enveloped small (8000 bp) DNA-virus with circular double-stranded DNA, showing a specific tropism for human epithelial cells both in the skin and mucous membranes [1, 2]. Since the 1970’s, evidence has been emerging causally linking HPV with different human neoplastic lesions. Until now, 200 HPV types have been fully characterized, comprising both i) cutaneous HPV types causing benign clinical manifestations known as skin warts (papilloma), ii) mucosal HPV types inducing benign papilloma, intraepithelial neoplastic and invasive cancer in the anogenital mucosa as well as in the respiratory (sinonasal, larynx, trachea, bronchus) and upper digestive tract (oral mucosa, oropharynx, esophagus) [3]. Of all HPV-associated malignancies, cervical cancer (CC) is undoubtedly the most important causing significant morbidity and mortality worldwide [3–5]. Given this plurality of HPV lesions in different anatomic sites, HPV types inducing asymptomatic or transient infections may use distinct strategies for transmission and propagation within the epithelium, and also for their interactions with the immune system [6].

HPV is the causal agent, necessary for the development of CC, and identified in practically all cases when highly sensitive detection methods are used. Cancer development is associated with HPV persistence that leads to cellular transformation, disease progression to precancerous lesions and, if uninterrupted, to an invasive cancer [7, 8].

HPV types infecting genital mucosa (in both genders) are subdivided into high-risk (hr) and low-risk (lr) groups. This classification is based on established epidemiological evidence on their association with benign, precancerous and cancer lesions. The hr-HPV are 16, 18, 31, 33, 35, 39, 45, 51, 52, 56, 58 and 59; whereas the lr HPVs include 6, 11, 40, 42, 43, 44, 54, 61, 70, 72 and 81. It is now obvious that both hr- and lr-HPV infections do occur in different anatomic sites, inducing either benign, premalignant or malignant lesions [3, 5, 6, 9].

### Human papillomavirus life cycle

The HPV life cycle is intimately linked with the programmed differentiation process of stratified squamous epithelia (cutaneous and mucosal), starting from the undifferentiated cells of the basal layer, capable of continuously dividing, and progressing through highly specific cellular events towards fully matured keratinocytes (on epithelial surface). In principle, HPV life cycle has three major phases. First, infectious virions access the basal layer through a micro-lesion, forming a virus reservoir where the viral genome is conserved as a low–copy number episomal form within the infected cell. Second, the viral genome replication occurs, whereby host cell DNA and the infected cells are divided into daughter cells, which can persist in the basal and parabasal layers and proceed to the division, or move and initiate the differentiation process. In the latter, activation of the late (L) gene expression and vegetative viral DNA amplification begin. This amplification leads to synthesis of a huge number of viral copies [6, 10, 11]. During hr-HPV infections, degradation of p53 and inhibition of pRb proteins by E6 and E7 viral proteins, respectively, promotes infected cell proliferation. These events are a critical determinant of the neoplastic grade, E6 and E7 expression levels being high in CIN 3 and low in CIN 1 lesions [4, 6, 12, 13].

### Recent insights into HPV infections

HPV infection is most easily transmitted in sexual intercourse, and the risk is higher in women with an early onset of sexual activity (due to higher exposure to the virus) as well as in those with impaired immunity [14–16]. Cervical intraepithelial neoplastic (CIN) and CC have been causally linked with HPV infection, which is considered the necessary prerequisite for the malignant transformation [17]. Virus entry is considered to take place in the basal cells of the squamous epithelium, specifically at the *cervical transformation zone (TZ),* where squamous epithelium meets the glandular epithelium of the endocervical canal. HPV-induced cell transformation is mediated by the viral E6 and E7 oncoproteins interfering with the cell cycle regulators of the host cells, starting at the basal cells layer and gradually extending to the full epithelial thickness [9, 18]. Once the infection is well established, the HPV genome is present as extra-chromosomal elements (episomes) in the nucleus, and the viral genome load is increased up to 50–100 copies per cell, while remaining high in the infectious phase [19, 20].

### Natural history of HPV infections

The great majority of sexually active women and men are likely to be infected by HPV at least once in their life-time [21]. In general, the vast majority of all HPV infections (90 %) are cleared without clinical diseases, spontaneously by means of immune response and do not persist long enough to cause oncogenic progression. Only about 10 % of HPV infections are expected to remain persistent [3, 21]. Individuals who develop benign lesions may also show regression mediated by the host immune response. About 50 % of the new infections are not detected before of 6–12 months and the majority of those will clear within 24 months. According to another concept on regression, the viral genome remains “latent” and may be reactivated by any trigger related e.g. to immune suppression or alterations of hormonal balance. When truly persistent, HPV infection bears a significantly increased risk for progression to high-grade lesion (CIN3) and CC during the years, if not adequately treated [3, 6, 9, 15, 16, 22].

Around 70 % of all CC cases are associated with hr-HPV 16 and 18, in contrast to benign genital warts, which are predominantly related to HPV 6 and 11. Of all hr-HPV genotypes, HPV 16 shows the highest probability for persistence, and thus has a major impact on the cancer risk. HPV 18 is the most frequent genotype found in adenocarcinomas [3, 6]. Up to 40 % of HPV infections are of mixed type, containing more than one HPV type. For this reason, the precise identification of the hr-HPV genotypes in HPV infections is important to disclose the woman at risk to develop CC. Despite the fact that also the majority of these infections clear spontaneously, persistent infections by oncogenic HPV are considered as a significant risk factor to CC [3, 21, 23].

### Cervical cancer incidence

**Fig. 1:**
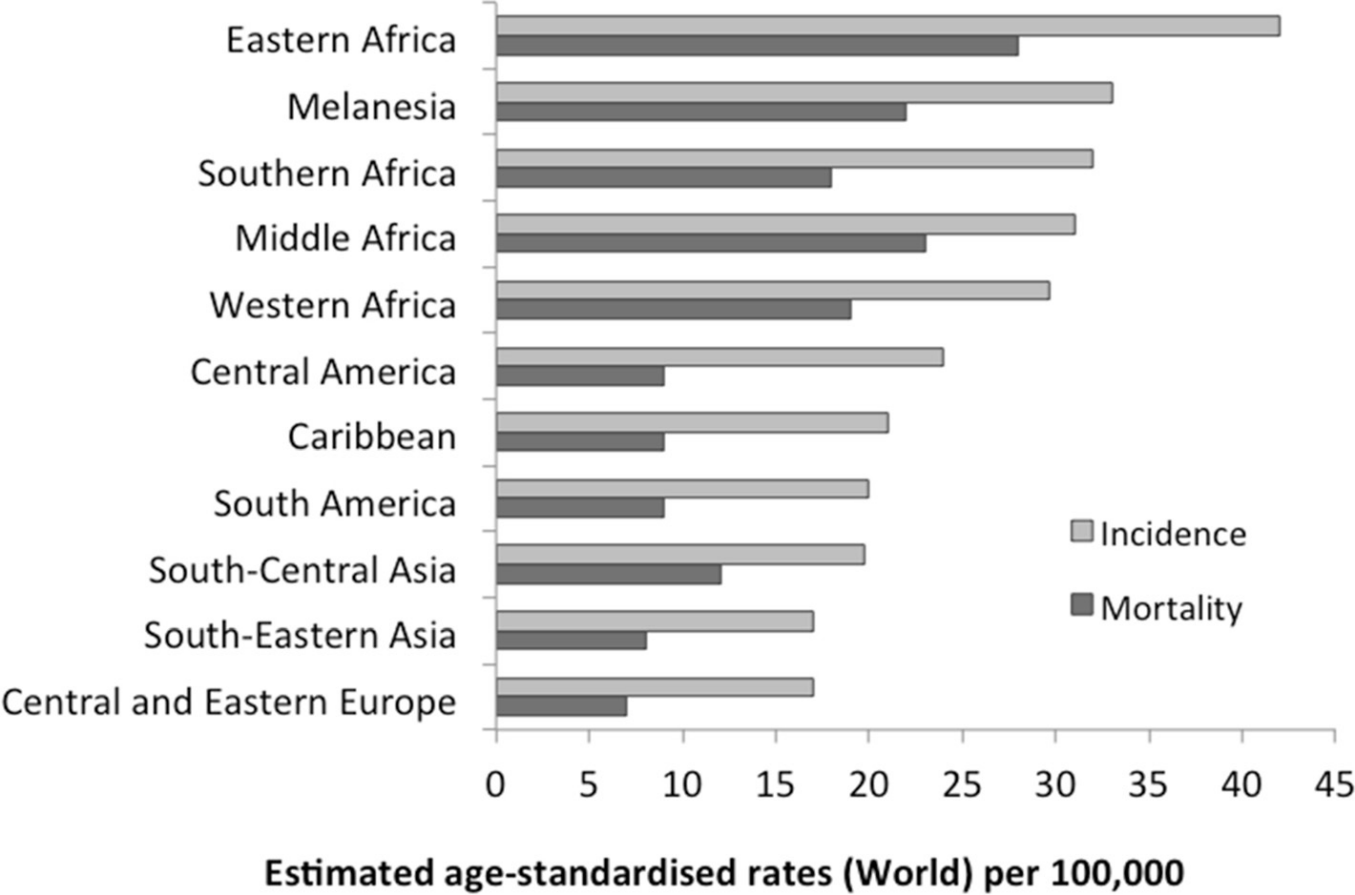
Global Incidence and Mortality of Cervical Cancer. Adapted from Globocan, 2012

**Fig. 2:**
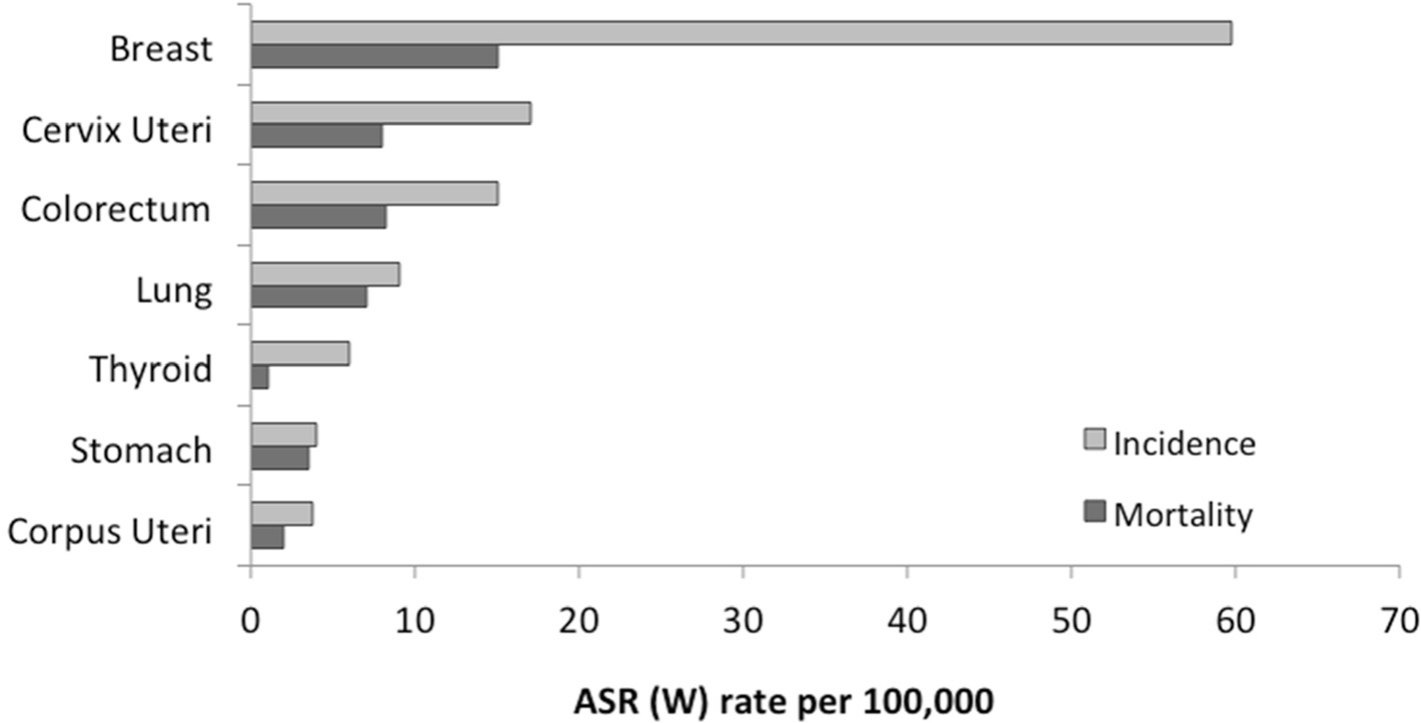
Estimated age-standardized incidence and mortality rates of Cervical Cancer in Brazilian population. Adapted from Globocan, 2012

According to the estimates for 2012, more than 528,000 incident cases and 266,000 deaths due to CC were detected worldwide [5]. About 85 % of these cases and 87 % total deaths occur in the developing countries, mainly due to poor access to health care for detection and treatment of early lesions [5]. The high-risk areas, with ASRs over 30 per 100,000 are Eastern Africa (42.7), Melanesia (33.3), Southern Africa (31.5) and Middle Africa (30.6) (Fig. 1). Brazilian figures comprise 24,500 incident cases and over 11,000 annual deaths (Fig. 2), with a mortality rate of 17 per 100,000 women [24–27].

The screening strategy in Brazil is targeted to women aged between 25 and 64 years, repeated every 3 years. Accordingly, the women should have Pap smear taken each three years if she has two consecutive normal smears in the previous screening rounds [28]. In the country, the mean age of detecting incident HPV infection, CIN3 and CC is about 20–, 30- and 40 years, respectively. The majority of the pre-malignant and malignant lesions affect squamous epithelium, where HPV types 16 and 18 predominate, the latter being relatively more important in glandular lesions (adenocarcinomas and their precursors) [14].

### HPV detection in preventing cervical cancer

It is established that an organized population-based screening and treatment of the detected precursor lesions can decrease the disease mortality by 80 %, as shown e.g. in Finland [3, 21]. CC progresses relatively slowly, and it takes years or even decades from an incident HPV infection to develop an invasive cancer. This slow process offers an opportunity to interfere with this cascade by detecting early precursor lesions and performs adequate therapy [3, 14, 21, 25, 29].

The first national initiative by the Brazilian government to implement a national screening program for CC was taken 40 years ago [30]. Since then, these programs have been improved and intensified, particularly during the past few years. A careful weighting is needed to optimize the program, i.e., to decide who are screened and when, based on a careful analysis between advantages and disadvantages, including the issues related to optimal cost-effectiveness.

Since its introduction, the cytological Papanicolaou smear (Pap test) has been considerate as the method-of-choice in organized screening for CC. When implemented as an organized program at population level, Pap smear screening has resulted in a substantial decrease of both incidence and mortality of CC [3, 21, 31]. In Brazil, there remains a lack of trained professionals with good skills in evaluating the Pap smears. Along with the huge territory, which represents another important obstacle, the scanty financial resources have been the limiting step in the full national implementation of Pap smear screening. Because of the same type of restrictions, most other developing countries continue showing high incidence and mortality rates of CC, in sharp contrast to many developed countries, with significantly reduced rates [21, 31, 32].

### CC prevention in Brazil

Brazil has not implemented an organized population-based CC screening program, and not even a universal system to invite all women to realize these examinations. However, the government has designed a program to identify and control the cytopathology examinations performed. The first examination used to identify CC precursor lesions is the Pap test, targeted to women from 25 years (sexually active) to 64 years, repeated every 3 years for women who had two consecutive negative smears within five years. However, the guidelines (national and international) do not recommend screening for women younger than 25 years by Pap test, mainly because most of these lesions clear spontaneously [30, 33].

Recognition that HPV infection is the necessary cause of CC opened new possibilities for prevention strategies, including the primary prevention with highly effective prophylactic HPV vaccines, and the secondary prevention using highly sensitivity HPV tests, which has significantly improved the performance of the screening programs based on the Pap test [33–35].

Molecular techniques have been considered better than cytology regarding the sensitivity and reproducibility in detecting CIN 2 or CIN 3. Proper treatment of the precursor lesions detected by screening should prevent the development of invasive CC and reduce the incidence and mortality. Some HPV tests used in screening have achieved a 60–70 % reduction in invasive CC incidence as compared with cytology alone [36]. Despite some test limitations, HPV testing can be a useful tool in the primary screening, including the self-sampling strategies. It has been demonstrated that baseline HPV-negative women have a lower risk to develop CIN and invasive CC if compared with women testing Pap smear negative. The most recent HPV test equipment and protocols are user-friendly procedures, easily reproducible in different settings. This is a major advantage, because many regions of Brazil are still lacking human resources, necessary to appropriately implement the Pap test, including qualified professionals and adequate quality control measures. However, the non-organized Brazilian program severely restricts the implementation of a systematic molecular screening by HPV tests that are already in widespread use in many developed countries. This implementation is hampered by the difficulties in confirming the diagnoses, to conduct follow-up and offer treatment of the detected cervical lesions [36–38].

During the past several years, a plethora of novel methods have been developed for the hr-HPV DNA testing in CC screening. The use of HPV DNA testing for oncogenic types has been proposed as a co-test with cytology for screening women above 30 years of age, showing higher accuracy as conventional Pap smear screening [17, 39–41]. Thus, HPV DNA testing could offer an effective measure to predict the risk of CC, suitable for implementation also in regions with lower resources [42]. Furthermore, self-collection of vaginal specimens for HPV testing could be useful particularly for women who live in regions with low-resource settings and restricted access to adequate health care, like in many regions of Brazil, thus increasing the acceptability and coverage of the screening program. However, HPV DNA testing is not yet a routine in Brazil, despite the fact that several studies in Brazilian population have demonstrated its potential usefulness in CC prevention [20, 43, 44].

The overall acceptance of HPV testing is higher among women who have experience on self-sampling (made at home), as compared with the physician collection [40]. Moreover, the self-sampling has lower cost and it is a truly non-invasive procedure. However, the gold standard of HPV testing still is the sampling by a physician (using speculum examination) [40, 45].

### Which type of screening for which population?

For almost 60 years now, cervical cytology (conventional Pap smear and more recently LBC), has been the gold standard in CC screening programs. Based on early detection of CC precursor (CIN) lesions, both tests represent measures of secondary prevention of CC. However, organized screening based on cervical cytology has severe limitations in implementation; extensive training is mandatory, good laboratory infrastructure is necessary, and standardization and quality control are essential [14, 42, 46].

The well-established natural history of HPV infections is the sound basis for the rational use of CC preventive measures based on HPV testing. The single most important goal of CC screening is to reduce mortality. This is achieved by decreasing the incidence of invasive disease by detecting the precancerous lesions, followed by their adequate treatment [9, 46, 47]. Ronco et all (2008) concluded that women aged 25–34 years, who test HPV+ in HPV DNA screening do not need an immediate referral to colposcopy, because of the high probability of spontaneous regression of even CIN 2 lesions in this age group. Combination of HPV DNA testing and cytology further decreases the rate of false-positive results, thus reducing the colposcopy referral rates [17, 48]. Similarly, one can expect a substantial decrease of false-negative results (the inherent problem of cytology) when HPV screening is used, which is another advantage with significant impact on screening efficacy.

### The vaccination and screening paradigm

Although HPV vaccination of teenagers and young women has proven effective in many countries, the public health strategies to demonstrate effective prevention of CC still represent major obstacles to overcome. The cost-effectiveness analysis for all strategies to reduce CC incidence should involve both the screening and HPV vaccination in an integrated and organized manner. At the moment, the preferable option in Brazil seems to be the use of HPV testing for primary screening, followed by triage of all HPV+ cases with the Pap test. A recent Mexican trial [49] showed a most marked improvement in CC prevention when HPV test was used in governmental program, which sounds feasible given the known limitations of the Pap test. Indeed, the expected reduction of high-grade lesions after widespread introduction of HPV vaccination is anticipated to further compromise the performance of Pap test [49, 50].

According to WHO, HPV types 16 and 18 are responsible for 70 % of all CC cases worldwide. The currently available vaccines against these two HPV types have the potential of reducing the incidence of cervical and anogenital cancer. Brazil has a population of >64 million women aged above 15 years and at risk to develop CIN and CC, who certainly would benefit from HPV vaccines [29]. In this country, National Health System adopted the quadrivalent HPV vaccine to the national vaccination program in 2014. The vaccine is offered for girls at 11–13 years of age. This age range was extended to cover girls aging 9 and 10 years, and the coverage includes more than 33,000 HIV positive women between 9–26 years [35].

With the widespread implementation of HPV vaccines, HPV infections and HPV-related lesions will decrease substantially, which might increase the rates of false-negative results in cytology, in addition to reduced specificity. The combination of HPV vaccination with HPV testing and cytological triage is suggested to be the optimal algorithm to protect women against CC [16, 34, 47].

## Conclusions

The most important applications of HPV testing include i) organized screening programs to optimize an early detection of HPV-induced CC precursor lesions, and, ii) to conduct adequate follow-up of the women who have been treated for high-grade CIN.

Some novel technical innovations of HPV testing are particularly designed for remote areas, without compromising the test performance for hr-HPV detection. Certainly self-sampling for HPV testing still needs standardization to make it compatible with the usual sampling techniques. Once successfully accomplished, it has a potential to become a valuable screening tool for large underprivileged populations, thus contributing to alleviating the significant disease burden in these countries.

## Abbreviations

ASR: Age standardized rate
CC: Cervical cancer
CIN: Cervical intraepithelial neoplasia
DNA: Deoxyribonucleic acid
HPV: Human papillomavirus
HPV+: HPV Positive
HR: High-Risk
IARC: International Agency for Research on Cancer
L: Later
LBC: Liquid-Based Cytology
LR: Low-Risk
TZ: Transformation zone
WHO: World Health Organization
